# Novel and known periodontal pathogens residing in gingival crevicular fluid are associated with rheumatoid arthritis

**DOI:** 10.1002/JPER.20-0295

**Published:** 2020-08-27

**Authors:** Daniel Manoil, Nagihan Bostanci, Gonca Mumcu, Nevsun Inanc, Meryem Can, Haner Direskeneli, Georgios N. Belibasakis

**Affiliations:** ^1^ Department of Dental Medicine Division of Oral Diseases Karolinska Institutet Stockholm Sweden; ^2^ Center of Dental Medicine University of Zürich Zürich Switzerland; ^3^ Faculty of Health Sciences Marmara University Istanbul Turkey; ^4^ Department of Internal Medicine Division of Rheumatology Faculty of Medicine Marmara University Istanbul Turkey; ^5^ Department of Internal Medicine Division of Rheumatology School of Medicine Medipol University Istanbul Turkey

**Keywords:** fluorescent in situ hybridization, gingival crevicular fluid, oral microbiology, periodontitis, Porphyromonas gingivalis, rheumatoid arthritis

## Abstract

**Background:**

Periodontitis is a suspected environmental risk factor for the development of rheumatoid arthritis (RA). However, correlation mechanisms between the two pathologies remain elusive. This study examined potential correlations between detached subgingival bacteria collected in gingival crevicular fluid (GCF) and RA parameters.

**Methods:**

RA patients (*n* = 52, F:M = 40:12), patients with Behcet's disease (BD, *n* = 40, F:M = 29:11) as another systemic inflammatory disease were studied along with a systemically healthy control group (HC, *n* = 57, F:M = 40:17). All participants were non‐smokers. Full mouth periodontal parameters were recorded. RA activity was assessed using the 28‐joint Disease Activity Score (DAS‐28). Rheumatoid factors (RFs)‐IgM and ‐IgA were measured by ELISA. GCF samples were investigated by means of fluorescent in situ hybridization for 10 different bacterial taxa.

**Results:**

The taxa TM7, *Synergistetes* cluster B, *Leptotrichia, Megasphaera*, *Anaeroglobus geminatus*, and *Tannerella forsythia* displayed significantly differential abundances between the groups. Whereas abundances of *Megasphaera* and *A. geminatus* were significantly increased in the RA group, only *Porphyromonas gingivalis* displayed significant correlations with plaque scores, bleeding on probing, and RF‐IgA. RA patients displaying RF‐IgA levels >75 IU/mL exhibited five‐fold more abundant *P. gingivalis* levels than patients below the threshold. This association with RF‐IgA levels appeared even more pronounced, by six‐fold more *P. gingivalis* (*P* = 0.025), in patients with a DAS‐28 score >3.2, indicative of moderate/very active RA.

**Conclusions:**

Unattached GCF bacteria may mediate the association between periodontitis and RA, and monitoring the bacterial composition of GCF might inform on RA activity. The role of newly identified bacterial taxa in RA warrants further investigations.

## 1 INTRODUCTION

Rheumatoid arthritis (RA) is a systemic inflammatory autoimmune disorder, characterized by chronic, erosive polyarthritis.^1^ It is the most common autoimmune disease affecting the synovium of the joints with a prevalence reaching up to 1% in the worldwide population.^1^ The disease causes chronic pain, disability and can further evolve towards more severe extra‐articular complications such as interstitial pneumonia or rheumatoid vasculitis.^2^ To date, the exact etiopathology of RA remains largely elusive. As with many other multifactorial autoimmune diseases it is postulated that, in genetically predisposed patients, various environmental factors may trigger a loss of immune tolerance in secondary lymphoid organs leading to the production of multiple autoantibodies, among which anti‐citrullinated protein antibodies (ACPA) and rheumatoid factors (RFs) are the most characteristic.^3^ Among several environmental factors studied for their potential contribution to the onset of RA, multiples lines of investigation point towards the involvement of periodontitis.^4^ Periodontitis is a biofilm‐driven oral inflammatory disease that affects between 15% to 47% of the worldwide population.^5^ The disease is initiated by biofilms that adhere onto subgingival teeth surfaces inducing a series of inflammatory reactions that lead to the destruction of tooth‐supporting tissues. It has been reported that periodontitis patients have twice the chance of developing RA, and that RA patients are almost twice as likely as patients with osteoarthritis to display moderate to severe periodontitis.^6^, ^7^ Moreover, certain biological immunomodulators administered for the treatment of RA were suggested to ameliorate the periodontal status, and more importantly, successful treatment of periodontitis was shown to decrease levels of RA markers and improve clinical outcomes.^8^, ^9^

One of the most substantiated hypothesis that underpins the association between RA and periodontitis suggests mucosal surfaces of periodontal pockets as sites of disease initiation.^10^ This postulate suggests that the dysbiotic microbiota of periodontitis generates some of the initial antigens that further cause the adaptive immune system to cross‐react with host epitopes.^10^ This has been mainly supported by the finding that *Porphyromonas gingivalis*, a recognized keystone pathogen associated with periodontitis,^11^ can catalyze the citrullination of bacterial and host proteins, and may therefore contribute to the generation of ACPAs.^12^ Nonetheless, recent studies on RA pathogenesis have also identified associations with other periodontal taxa.^13^, ^14^ For instance, several *Prevotella* species were detected in increased abundances in RA patients independently of their periodontal status.^14^ The genera *Prevotella* and *Leptotrichia* have also been associated with new‐onset RA.^13^ The species *Anaeroglobus geminatus* was further reported to positively correlate with ACPAs and RFs in RA‐patients.^13^ Overall, studies correlating subgingival microbiotas with RA are characterized by considerable variations. The difficulty to establish causal relationship between periodontitis and RA lies in good part within the polyfactorial nature of RA and the important heterogeneity of its clinical presentation and progression. One approach to mitigate such heterogeneity may include the rigorous recruitment of a RA cohort that displays homogenous disease characteristics and limits the influence of other environment risk factors, such as smoking. Furthermore, concentrating analyses on cases that exhibit more severe presentations of RA may also contribute to the attenuation of the effect of unmeasured confounding factors.

The gingival sulcus or periodontal pocket appears as unique niches of the oral cavity in that they are bathed by gingival crevicular fluid (GCF).^15^ The conformation of the sulcus or pocket enables a “loosely adherent” plaque zone that can be collected at the orifice of the pocket by means of Periopaper strips.^15^, ^16^ GCF as a serum transudate or inflammatory exudate can markedly affect the composition of the bacterial communities that colonize this niche.^17^ In contrast to the well‐studied composition of subgingival plaque in patients with RA, no previous knowledge exists of the GCF bacterial composition.^18^ It is plausible that bacteria detached from subgingival biofilms into the GCF may find their way into the gingival microcirculation via the ulcerated gingival epithelium, thus contributing to systemic inflammation.^17^

Therefore, we aimed to identify potential correlations between detached subgingival bacteria in GCF, and serum levels of RFs. These investigations were conducted on a cohort of RA‐affected patients using systemically healthy participants as controls and Behçet disease patients, another systemic inflammatory disease associated with poor oral health. Specifically, a range of 10 selected bacterial taxa was quantified from GCF samples by fluorescent in situ hybridization (FISH) or immunofluorescence (IF). Bacterial abundances were then compared and correlated with RFs‐IgM and ‐IgA measured from serological samples by ELISA. Particular focus was finally laid onto more severe cases of RA, narrowed down using serological levels of RFs, and the 28‐joint Disease Activity Score.^19^

## 2 MATERIALS AND METHODS

### 2.1 Description of the study cohort

The current cross‐sectional study included 149 participants recruited at the Division of Rheumatology, School of Medicine, Marmara University, Turkey, between November 2010 and April 2012. This cohort was composed of three groups; RA patients (*n* = 52, F/M: 40/12), Behçet's disease patients (BD, *n* = 40, F/M: 29/11) and healthy control participants devoid of systemic inflammatory disorders (HC, *n* = 57, F/M: 40/17). The study conformed to provisions of the Declaration of Helsinki and was approved by the Ethical Committee of Marmara University Medical School (MAR‐YÇ‐ 2009‐0295). All patients included gave their written informed consent.

RA‐affected patients were clinically diagnosed according to the classification criteria of the 1987 American College of Rheumatology (ACR).^20^ All RA patients were under conventional synthetic disease‐modifying anti‐rheumatic drugs (csDMARDs) treatment for >6 months, yet never received biologic therapy. RA activity was assessed using the 28‐joint Disease Activity Score (DAS‐28).^19^ BD‐affected patients were diagnosed according to the classification criteria implemented by the International Study Group of Behçet disease and were recruited herein to serve as an inflammatory disease group associated with oral health impairment.^21^, ^22^ Participants included in the HC group were matched for gender and socio‐economic status. HC participants were selected among family‐unrelated individuals devoid of any systemic inflammatory disorders who accompanied patients attending the Rheumatology outpatient clinic. Patients with cardiovascular and respiratory diseases, diabetes mellitus, immunosuppressive therapy or currently pregnant or lactating were excluded from this study. Likewise, participants who had taken antibiotics or other medication recognized to potentially affect their oral microbiota and/or periodontal status within 6 months preceding their enrollment were also excluded. All participants were non‐smokers.

### 2.2 Measurement of periodontal parameters and collection of GCF

At enrollment, all participants were examined by a specialist dentist in Oral Medicine (GM). Their periodontal status was evaluated by means of radiographic and clinical parameters that included a full mouth probing pockets depth, clinical attachment loss (CAL), bleeding on probing (BoP), and a Quigley‐Hein plaque index (PI) at six sites around each tooth with a manual probe.^23^ All teeth were included in the examination except third molars.^24^ Participants displaying ≥2 interproximal sites with CAL values ≥4 mm (not on the same tooth) were defined as periodontitis positive.^24^

For GCF collection, supragingival plaque was first removed from the collection sites, which were then isolated with cotton rolls and gently dried by air blowing. A sterile Periopaper strip* was then placed at the orifice of the periodontal pocket for 30 sec. Caution was taken not to mechanically irritate tissues or to cross‐contaminate the strip with supragingival tooth surfaces or saliva. GCF was collected from the mesio‐buccal sites of the four deepest periodontal pockets/sulci in each quadrant. Four Periopaper strips were therefore generated for each participant unless they had no teeth in one or several quadrants. GCF samples were stored at ‐80°C until further processing.

### 2.3 Blood sampling

All participants provided fasting peripheral blood. Blood samples were allowed to clot at room temperature and were then centrifuged for 15 min at 1500 × *g* – 4°C to remove the fibrin coagulate and cellular elements. Aliquots were measured for RF‐IgM and RF‐IgA by ELISA.† For RF‐IgM, intra‐, and inter‐assay variabilities ranged from 4.7% to 7.4% and from 6.7% to 12.2% respectively. For RF‐IgA, intra‐, and inter‐assay variabilities ranged from 2.5% to 11.2% and from 4.1% to 16.8% respectively. Thresholds used to define positivity and high‐level positivity relied on the classification criteria of the 2010 American College of Rheumatology (ACR) / European League Against Rheumatism.^25^ Based on the upper limit of normal (ULN) for the respective laboratory assay, the following definitions were established; negative values are ≤ to the ULN; low‐level positive values are > than the ULN but ≤3 times the ULN and high‐level positive values are ≥3 times the ULN for that laboratory assay.^25^ Accordingly, RF‐IgM levels >10 IU/mL and RF‐IgA levels >25 IU/mL were required for positivity. Highly positive RFs measurements were further used to narrow down more severe cases defined by RF levels three fold above the positivity threshold, i.e., 30 IU/mL and 75 IU/mL for IgM and IgA isotypes respectively.

### 2.4 Quantification of bacteria by fluorescent in situ hybridization and immunofluorescence

Representatives of the domain Eubacteria along with nine selected periodontal taxa were detected and quantified by means of FISH or IF. Eight FISH oligonucleotide probes labeled with Cy3 or FAM were employed for the labeling of Eubacteria, *Prevotella intermedia, Synergistetes* cluster A, *Synergistetes* cluster B, *Leptotrichia, Anaeroglobus geminatus, Megasphaera*, and TM7. Accordingly, IF was employed for *Porphyromonas gingivalis* and *Tannerella forsythia*. On the day of analysis, the strips were pooled together into 1.5 mL tubes to form one GCF sample per participant. GCF was eluted from the strips into phosphate‐buffered saline containing protease inhibitors (PBS, pH 7.4) at 4°C by overnight gentle shaking. After centrifugation at 5000 rpm for 10 minutes, the supernatants were removed and the cellular pellet was suspended into NaCl 0.9% with RNase inhibitor.‡ This bacterial suspension was then transferred into fresh 1.5 mL tubes, and processed for FISH or IF onto multi‐well epoxy coated slides§ (4 mm diameter wells, 10 µL each) as previously described.^26^, ^27^ Table S1 in online Journal of Periodontology lists the oligonucleotide probes and antibodies employed in this study along with the references in which they have been validated.

Counting of labeled bacteria from multi‐well slides was performed under a fluorescence microscope.** Fluorescence was collected using the filter sets U‐MNIBA (6‐FAM), U‐MA41007 (Cy3), and BX‐DFC5 (6‐FAM / Cy3). Field of views (FoVs) to be assessed were selected blindly. To ensure that bacterial counts reached between 80 and 300 cells, the number of FoVs counted was adjusted to the density of labeled bacteria. At high cell density, i.e.,  >20 labeled bacteria per FoV, from two to five FoVs were counted. Alternatively, at intermediate densities, up to 48 FoVs were counted, whereas at low densities, i.e, <1 labeled bacterium per FoV, the entire well was systematically assessed. Photomicrographs were acquired using an incorporated camera.†† Representative images presented in Figure 2 were adjusted using ImageJ 1.5m9 for clarity purposes (brightness, contrast, and background subtraction), without altering the image quantitative or qualitative content. Results are expressed as the number of specific bacterial taxa normalized to four Periopaper strips, as four quadrants were potentially sampled.

### 2.5 Statistical analyses

Statistical analyses were performed using SPSS software version 25. Kruskal‐Wallis tests were employed when comparing the three groups among them and Mann‐Whitney tests were employed to compare taxa between two groups. Correlations were identified by Spearman coefficients. *P* values below 0.05 were considered statistically significant.

## 3 RESULTS

### 3.1 Clinical and demographic findings

Table 1 provides an overview of the clinical characteristics of the study cohort. No gender differences were observed among the three groups (*P* > 0.05) with females consistently representing around 73% of each group. The mean age was higher in RA patients than in BD and HC participants (*P* = 0.000). Disease duration was similar in RA (8 ± 7.2 years) and BD (10 ± 6.3 years) groups (*P* > 0.05). RA patients displayed a mean DAS‐28 score of 4.5 ± 1.6. All RA patients were treated by csDMARDs for > 6 months, excluding biologic agents.

**TABLE 1:** Overview of the clinical characteristics of the study cohort along with the distribution of the site‐specific periodontal parameters (i.e., corresponding to the sites of microbial sampling)

Characteristics	RA (*n* = 52)		BD (*n* = 40)		HC (*n* = 57)		Pair of groups comparisons	
SEX	n	%	n	%	n	%		
Female	40	77	29	72.5	40	70.2		
Male	12	23	11	27.5	17	29.8		
CLINICAL PARAMETERS	Mean	SD	Mean	SD	Mean	SD		
Age (years)	48.04	10.26	37.67	9.28	34.95	9.87		
Disease duration (years from diagnosis)	7.96	7.24	10.03	6.29	/	/		
DAS‐28	4.51	1.62	/	/	/	/		
PERIODONTAL STATUS	n	%	n	%	n	%	RA – BD	RA – HC
Periodontitis ‐	37	71.15	31	77.5	53	92.98	0.494	**0.003**
Periodontitis +	15	28.85	9	22.5	4	7.1		
SITE‐SPECIFIC PERIO.	Median	IQR	Median	IQR	Median	IQR	RA – BD	RA – HC
PPD	2.9	3.6–2.6	3	3.3–2.7	2.9	3.5–2.3	0.794	0.539
CAL	3.6	4.2–2.7	3.3	3.8–2.6	3.5	3.9–2.7	0.221	0.410
PI (Q‐H)	0.9	2.2–0.3	0.5	1.8–0.1	0.6	1.1–0	0.082	**0.006**
BoP	25	100–1.3	23.9	87.5–0	11	75–0	0.587	0.147

On the right side of the table, pair of groups comparisons display *P* values of Mann‐Whitney tests (*P* = 0.05).

Significant *P* values are bolded. BD, Behçet disease; BoP, bleeding on probing; CAL, clinical attachment loss; DAS‐28, 28‐joint disease activity score; HC, healthy controls; IQR, interquartile range (Q3‐Q1); PI (Q‐H), plaque index (Quigley‐Hein); PPD, probed pocket depth; RA, rheumatoid arthritis

The prevalence of periodontitis was higher in the RA group (28.9%) compared to BD (22.5%) and HC (7.1%) groups (*P* = 0.012) (Table 1). Periodontal parameters corresponding to the sites of GCF collection, i.e., site‐specific periodontal parameters, were rather equally distributed among participants of the four groups (*P* > 0.05), except for PI (*P* = 0.018). Indeed, paired group comparisons showed that RA patients exhibited a significantly higher PI, compared to HC participants (*P* = 0.006), whereas no differences were observed between RA and BD groups (Table 1).

### 3.2 Microbiological findings

**FIGURE 1:**
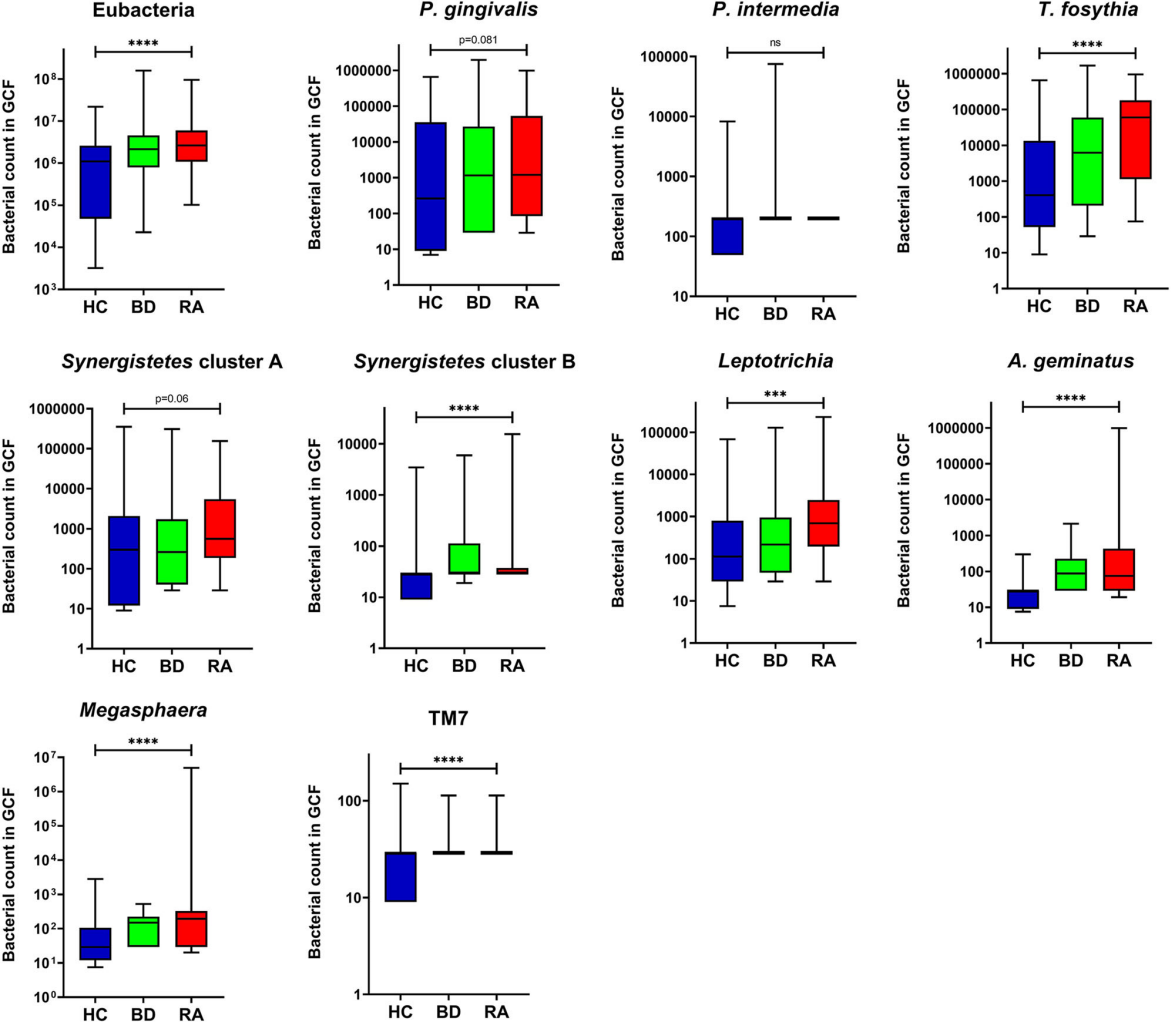
Distribution of the selected bacterial taxa between the three groups; healthy controls (HC), Behçet disease (autoimmune mediated‐disease patients) and RA (rheumatoid arthritis patients) (Kruskal‐Wallis, *P* = 0.05). Boxplots show the first and third quartile (top and bottom edges of the rectangle) divided by the median. Whiskers correspond to the highest and lowest values. *** *P* ≤ 0.001, **** *P* ≤ 0.0001

**FIGURE 2:**
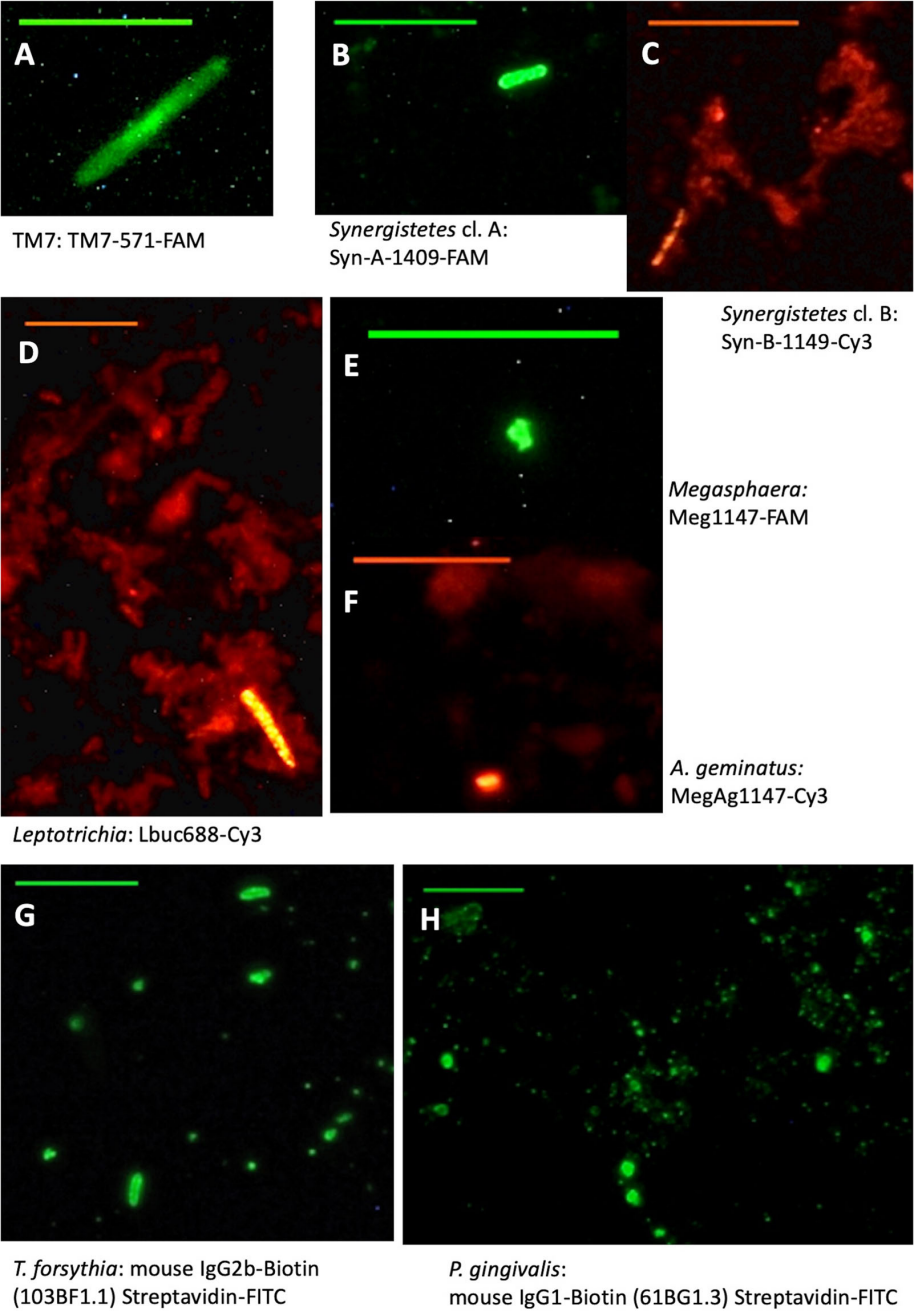
Representative photomicrographs of fluorescent in situ hybridization (FISH) and immunofluorescence (IF) labeling of GCF samples. Linked to each subfigure is a description of the corresponding taxon and of the FISH oligonucleotide probes, alternatively IF antibodies, utilized. All scale bars represent 10 µm

The combinational use of an Eubacteria FISH probe along with nine taxonomically more specific probes and antibodies revealed a differentially distributed microbiota in the GCF of the 3 groups (RA, BD, and HC) (*P* < 0.05) (Figure 1). More specifically, the phyla TM7 (Figure 2A) and *Synergistetes* cluster B (Figure 2C) along with the genera and species *Leptotrichia* (Figure 2D), *Megasphaera* (Figure 2E), *A. geminatus* (Figure 2F), and *T. forsythia* (Figure 2G), all displayed significantly differential abundances (*P* < 0.05) between groups. The taxa *Synergistetes* cluster A (*P* = 0.06) (Figure 2B) and *P. gingivalis* (*P* = *P* = *P* = 0.08) (Figure 2H) showed *P* values bordering significance, whereas *P. intermedia* showed no significant abundance differences between groups (Figure 1). Abundances of *Leptotrichia*, *Megasphaera*, *A. geminatus*, and *T. forsythia* were significantly increased in the RA group (*P* < 0.05).

Further comparisons of the bacterial distribution between pair of groups showed that the increased abundances of the taxa *Leptotrichia* and *T. forsythia* in RA as compared to BD were responsible for distinguishing these two groups (*P* < 0.05) (Table 2). Comparisons between RA and HC showed that all taxa, aside from *P. intermedia*, appeared differentially distributed and most frequently displayed increased counts in RA patients (*P* < 0.05) (Table 2). Additional comparisons between BD and HC are provided in Table S2 in online Journal of Periodontology.

**TABLE 2:** Comparison of the bacterial distribution between pair of groups (Mann‐Whitney, *P* = 0.05)

Bacterial taxa	Groups	Median	SEM	Mean rank	*P*	Groups	Median	SEM	Mean rank	*P*
Eubacteria	RA	2′650’000	1′901’000.3	48.96	0.313	RA	2′650’000	1′901’000.3	67.82	**0.000**
	BD	2′145’000	4′241’598.4	43.3		HC	1′100’000	452′066.6	43.31	
*P. gingivalis*	RA	1′205	28′039.2	47.62	0.645	RA	1205	28039.2	61.44	**0.041**
	BD	1′160	58′871.7	45.05		HC	263	18′555	49.12	
*P. intermedia*	RA	199	0	46	0.254	RA	199	0	58.5	0.172
	BD	199	1′870	47.15		HC	199	224.2	51.81	
*T. forsythia*	RA	60′000	33′938.9	51.64	**0.035**	RA	60′000	33′938.9	69.57	**0.000**
	BD	6′205	48′143.9	39.81		HC	405	16′401.8	41.71	
*Synergistetes* cl. A	RA	563	4′898.3	50.40	0.109	RA	563	4898.3	61.89	**0.029**
	BD	263	10′425	41.43		HC	300	6313.7	48.71	
*Synergistetes* cl. B	RA	29	297.1	45.41	0.585	RA	29	297.1	67.15	**0.000**
	BD	29	194.4	47.92		HC	29	60.2	43.91	
*Leptotrichia*	RA	687.5	4′498.1	52.29	**0.018**	RA	687.5	4′498	66.85	**0.000**
	BD	219	4314.8	38.98		HC	113	1′369.2	44.19	
*A. geminatus*	RA	75	19′033.8	47.44	0.689	RA	75	19′033.8	70.37	**0.000**
	BD	87	52.5	45.28		HC	29	8.4	40.98	
*Megasphaera*	RA	194	95′186.2	50.16	0.128	RA	194	95′186.2	70.4	**0.000**
	BD	150	20	41.74		HC	29	49.7	40.95	
TM7	RA	29	1.8	47.13	0.462	RA	29	1.8	64.51	**0.000**
	BD	29	2.1	45.68		HC	29	2.8	45.21	

The left part of the table compares RA to BD patients and the right part RA patients to HC participants.

Significant p values are bolded. BD, Behçet disease; HC, healthy control; RA, rheumatoid arthritis; SEM, standard error of the mean

### 3.3 Correlation of microbial taxa with site‐specific periodontal parameters

**FIGURE 3:**
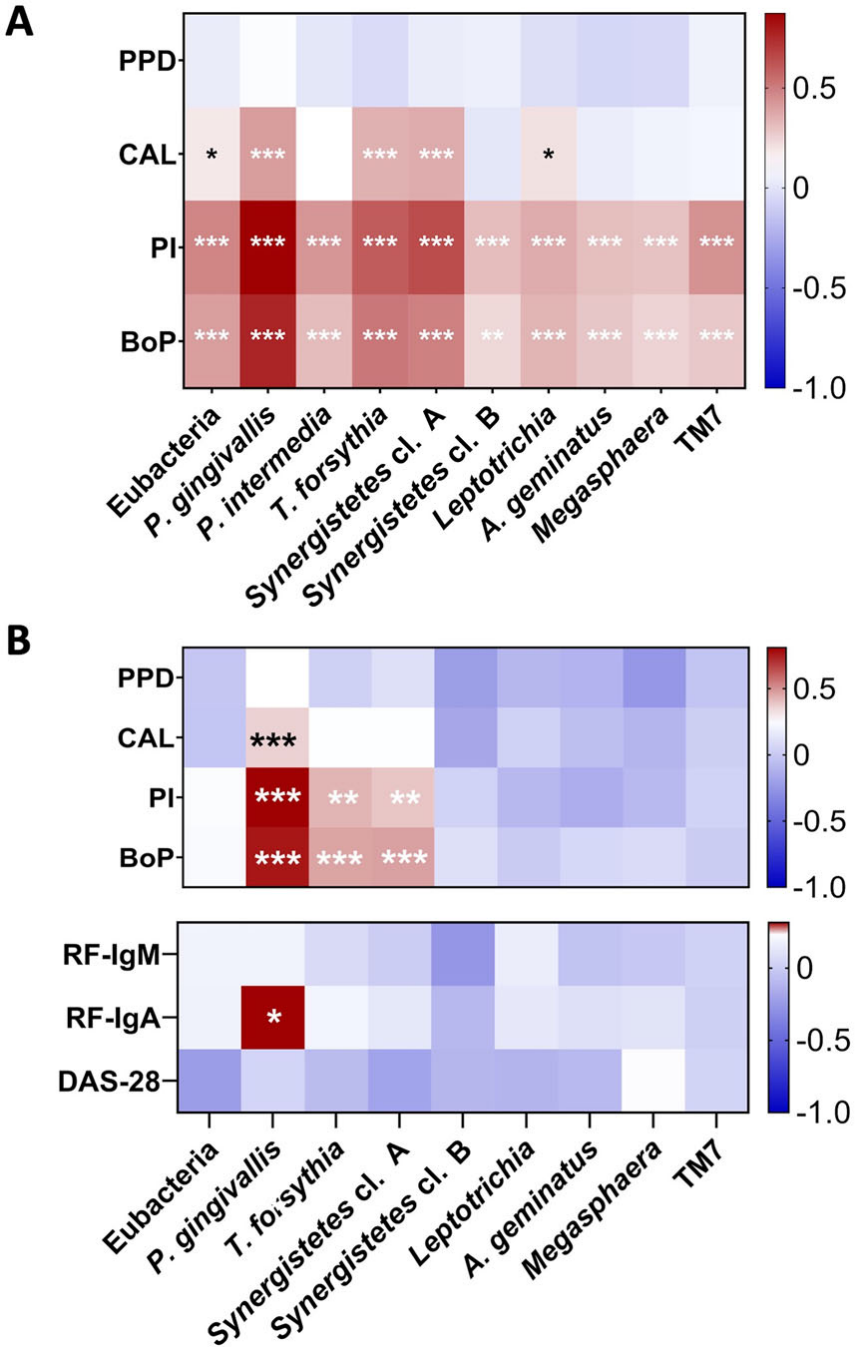
Heatmaps illustrating Spearman's correlations. **A**: correlations among the three groups (RA, BD, and HC) between the selected bacterial taxa and site‐specific periodontal parameters. **B**: correlations within the RA group between the selected bacterial taxa, periodontal parameters, serological rheumatoid factors, and the Disease Activity Score 28 (DAS‐28). The taxon *P. intermedia* could not be plotted here because its abundance did not vary among patients of the RA group. Significant correlations are flagged as: *P ≤ 0.05, ** *P* ≤ 0.01, *** *P* ≤ 0.001. BoP, bleeding on probing; CAL, clinical attachment loss; DAS‐28, 28‐joint Disease Activity Score; PI, plaque index (Quigley‐Hein); PPD, probed pocket depth; RF, rheumatoid factor

Correlation analyses among the three groups showed positive correlations between all taxa investigated and site‐specific periodontal parameters, mostly with PI and BoP (Figure 3A). The taxa *P. gingivalis*, *T. forsythia*, and *Synergistetes* cluster A in particular exhibited higher correlation coefficients (rho >0.5) with these two periodontal parameters (Figure 3A). In the RA group more specifically, *P. gingivalis* further displayed strong positive correlations with PI (rho 0.81, *P* = 0.000) and BoP (rho 0.76, *P* = 0.000) (Figure 3B).

### 3.4 Correlation of microbial taxa with systemic RA parameters

Correlations with RFs revealed that *P. gingivalis* significantly correlated with RF‐IgA (rho 0.32, *P* = 0.02) but not with RF‐IgM (rho 0.18, *P* = 0.19). No correlations could be established with *P. intermedia* because abundance of this species did not vary in the RA group and consistently displayed counts <200 cells in the GCF samples.

**FIGURE 4:**
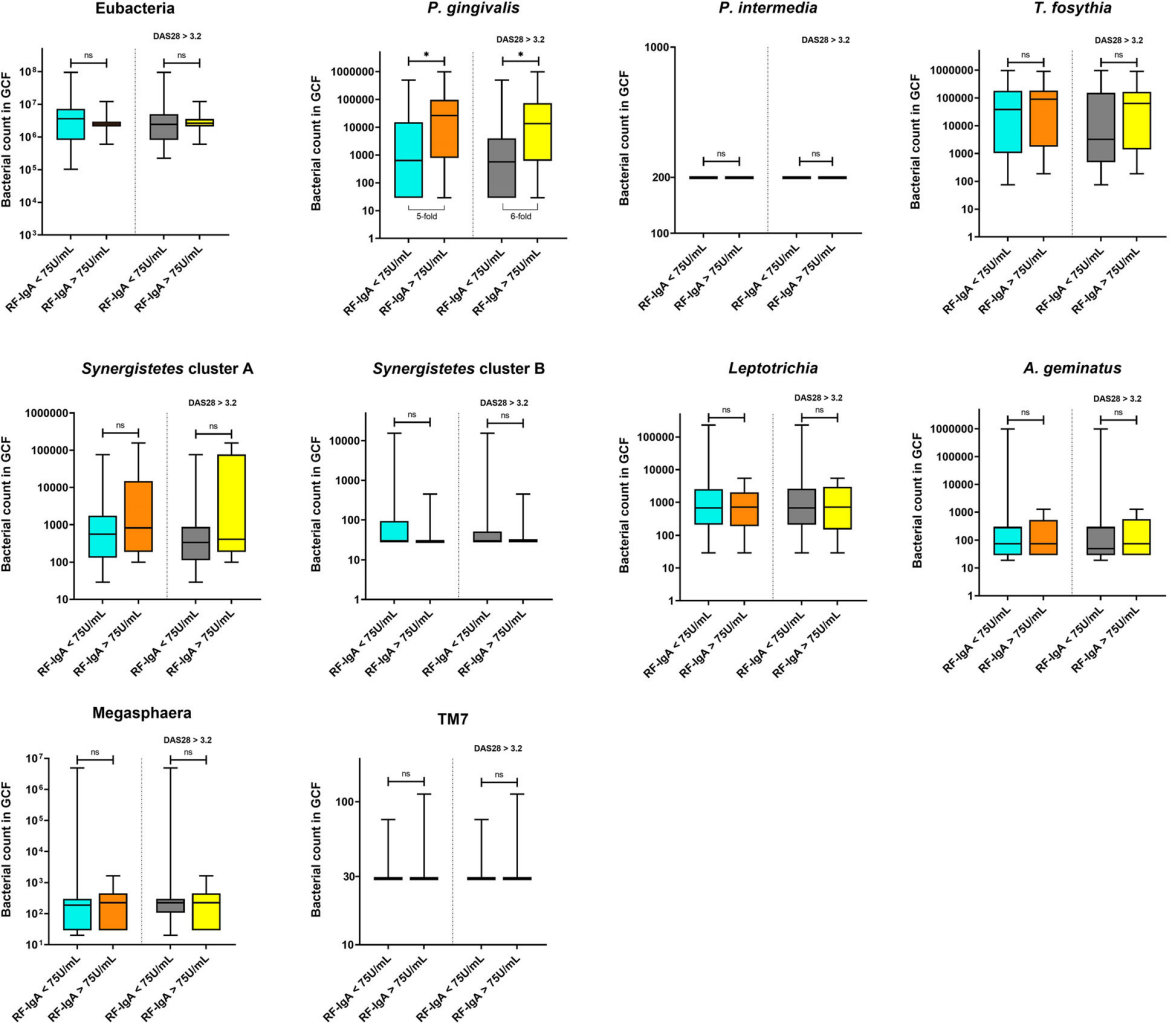
Compared abundances of the selected bacterial taxa in severe cases of RA. The left part of each plot compares bacterial abundances between patients presenting RF‐IgA levels < 75 IU/mL and > 75 IU/mL) (Mann‐Whitney, *P* = 0.05). The right part of each plot compares bacterial abundances according to RF‐IgA levels in patients, who additionally displayed a DAS‐28 score > 3.2, indicative of moderate/very active RA (Mann‐Whitney, *P* = 0.05). Boxplots show the first and third quartile (top and bottom edges of the rectangle) divided by the median. Whiskers correspond to the highest and lowest values. DAS‐28; 28‐joint Disease Activity Score, RF; rheumatoid factor

We further analyzed correlations between the taxa investigated and RFs in more severe cases of RA, which were defined as cases presenting RF levels three‐fold above the positivity threshold (RF‐IgM; 30 IU/mL, RF‐IgA; 75 IU/mL). In the case of RF‐IgM, no correlations appeared between RF‐IgM levels and the bacterial taxa investigated, despite the increase in threshold (data not shown). However, we observed that RA patients exhibiting RF‐IgA levels >75 IU/mL (*n *= 15) exhibited five‐fold more abundant *P. gingivalis* than patients below this threshold (*P* = 0.019) (Figure 4). In addition to RF‐IgA levels >75 IU/mL, we then attempted to sort patients who displayed a DAS‐28 score >3.2, indicative of moderate/very active RA. In this small cluster of patients, with both RF‐IgA >75 IU/mL and DAS‐28 >3.2 (*n* = 2), the association of *P. gingivalis* with RF‐IgA levels appeared even more pronounced, by six‐fold greater *P. gingivalis* levels (*P* = 0.025) (Figure 4). Evidently, this last analysis remains purely indicative for the time being, because too few patients displayed these cumulative parameters to provide statistical relevance.

## 4 DISCUSSION

Based on a combination of several taxonomic FISH probes and IF antibodies, results from the current report showed that RA‐affected patients exhibit a GCF microbiota composition different from BD and HC groups. Our results further indicated that the species *P. gingivalis* positively correlated with serological and clinical indicators of disease severity in patients with RA.

In this study, microbial identification was performed on cells recovered from strips placed at the orifice of periodontal pockets that primarily collect planktonic bacteria detached from the mass of subgingival biofilms.^17^ Detachment of bacteria from biofilms is part of their biofilm life cycle and is accompanied by an upregulation of genes involved in virulence and host cell invasion.^28^ These detached bacteria are challenging the integrity of the pocket epithelium and interacting with the adaptive immune system.^29^ Because such dysbiotic interactions are the suspected trigger that leads to immune cross‐reactivity, GCF bacteria appear particularly relevant to the etiopathogenesis of RA. Here, seven of the taxa investigated in GCF were differentially abundant between RA‐affected patients, HC, and BD participants, indicating that RA may be associated with an altered GCF microbial composition. Notably, average counts of the taxa *Leptotrichia* along with and *A. geminatus* and *Megasphaera*, which are two closely related members of the Veillonellaceae family, were significantly increased in the RA group. These findings are in line with a previous report by Lopez‐Oliva et al., which also identified *Leptotrichia* and Veillonellaceae members among taxa significantly enriched in RA patients.^18^ Furthermore, similar observations were reported by Scher et al. who detected the taxon *Leptotrichia* as characteristic of patients with new‐onset RA irrespective of their periodontal status.^13^ The same authors also reported increased abundances of *A. geminatus* in RA patients, and further identified positive correlations with ACPAs and RFs.^13^ Although the lack of literature on *A. geminatus* renders interpretation of these common findings difficult, an in vitro report has demonstrated that *A. geminatus* impacts the protein expression and functional properties of other species to potentially enhance the community's virulence.^30^ An indirect influence of *A. geminatus* on RA pathogenesis through shifts in the ecology of the periodontal pocket could therefore be speculated, yet remains to be investigated.

One bacterial species that may appear lacking from current analyses is *Aggregatibacter actinomycetemcomitans*. Indeed, recent evidence has shown that the leukotoxin (LtxA) of *A. actinomycetemcomitans* induces neutrophil hypercitrullination, resulting in a spectrum of citrullinated proteins detected in GCF that closely resembles the one detected in the synovial fluid of RA patients.^31^ The pre‐selection of probes and the limited number of taxa investigated reflects the close‐ended analytical nature of FISH, which may be perceived as a limitation compared to open‐ended high‐throughput analyses. Nonetheless, the periodontitis patients of this cohort are classified with a “chronic periodontitis”’ diagnosis, where *A. actinomycetemcomitans* is not a frequent find. In contrast, high LtxA–producing *A. actinomycetemcomitans* strains remain mostly associated with localized aggressive periodontitis, which association with RA, thus far, remains unclear.^32^

In this work, site‐specific periodontal parameters were used for correlation analysis, i.e., corresponding to sites of microbial sampling, rather than aggregation of full‐mouth site‐based data at the patient level. Indeed, consideration of site‐specific periodontal measurements allows for accurate correlations to be drawn with FISH microbial observations stemming from the corresponding GCF collection sites. More importantly, whereas potential periodontal risk factors were present long before the onset of RA, many of these patients presented with teeth loss. Full‐mouth average periodontal values in these analyses may therefore not have been representative of the periodontal history and existing risk factors preceding RA. Positive correlations between site‐specific PI and BoP were identified with all taxa examined. Because increased bacterial numbers, biofilm formation(PI), and gingival inflammation (BoP) are interconnected, these findings are coherent.^33^ In addition, *P. gingivalis*, *T. forsythia*, and *Synergistetes* cluster A significantly correlated with CAL values. Although *P. gingivalis* and *T. forsythia* are recognized members of the classical “red complex” associated with progressive tissue destruction, several studies also showed associations between *Synergistetes* cluster A and periodontal lesions.^34^, ^35^, ^36^ In addition, *P. gingivalis* levels in GCF are shown to correlate well with the corresponding levels of inflammatory mediators in the same milieu.^37^

Whereas previous studies classically investigated putative association mechanisms between periodontal taxa and the presence of ACPAs, the current report examined correlations with RFs.^12^, ^38^ RFs are a family of autoantibodies directed against the Fc fragment of IgG's.^39^ Detection of high titers and high affinity RFs in established RA represents a recognized severity factor associated with extra‐articular manifestations, which is not the case for ACPAs.^40^, ^41^ Increased serological levels of RF‐IgA more particularly, were shown to be strongly associated with joint erosion, complications outside joints and thus poorer prognosis.^39^, ^42^ Remarkably, of the 10 different taxa examined in this report, *P. gingivalis* was the only species to significantly correlate with RF‐IgA, suggesting a potential association of this taxon with more severe RA. This assumption was further supported by current observations indicating that *P. gingivalis* was detected in five‐ to six‐fold increased abundances in patients with elevated RF‐IgA levels and presenting an active RA (DAS‐28 >3.2). These findings complement and extend previous reports that have suggested *P. gingivalis* to play a role in the expression of RFs. Indeed, an early study by Gargiulo et al. detected RFs in subgingival plaque and inflamed gingival tissues from periodontitis‐affected patients, who also were more likely to be RF‐seropositive than oral healthy controls.^43^ More recently, Mikuls et al. examined the relationship between serological levels of anti‐*P. gingivalis* antibodies and the presence RFs in individuals at risk for RA. They reported that for each log‐fold increase in anti‐*P. gingivalis* antibody concentration, participants were 40% to 70% more likely to be RF‐seropositive.^44^ Because the Fc fragment of IgG's contains lysin and arginine residues, it has been purported that lysin‐ and arginine‐gingipains expressed by *P. gingivalis* may alter this region thereby enhancing binding of RFs.^45^, ^46^ Besides, recent evidence indicates that RFs recognize cryptic epitopes on the Fc fragment, different for IgM's or IgA's, that seem exposed only after a conformational change of the IgG induced upon antigen binding.^47^ This may somewhat afford an explanation to why only RF isotype IgA correlated with *P. gingivalis*.

It is finally worth mentioning that no RA patient recruited in this study was RA treatment naïve, even though caution was taken to only include patients under csDMARDs and exclude those under biological therapies. Although ethically unavoidable in RA patients, some csDMARDs, such as methotrexate for instance, were shown to significantly decrease serum levels of RF‐IgM and ‐IgA, thereby potentially underestimating the full magnitude of some correlations in this study.^48^ Such immunomodulatory medication may also be suspected to impact the GCF microenvironment and its microbial communities by modulating periodontium inflammation. However, evidence supporting this consideration remains contrasting.^49^ Although csDMARDs were shown to ameliorate the gains of non‐surgical periodontal treatment in RA patients, they appeared not to influence the gingival index or BoP, as compared to baseline or controls free of RA.^49^ Another study showed that a 3‐month intake of non‐steroid anti‐inflammatory drugs (NSAIDs), also potentially prescribed in RA, was unlikely to significantly affect the bacterial composition of subgingival plaque.^50^ Although these reports tend to suggest that the intake of csDMARDS or NSAIDs by RA patients in the current study was unlikely to have significantly impacted the GCF bacterial composition, such effect could not be definitely ruled out.

## 5 CONCLUSION

In conclusion, the present results indicate that seven of the nine periodontal taxa examined were differentially abundant in the GCF of RA patients compared to control groups. Notably, increased abundances of *Leptotrichia*, *Megasphaera*, and *A. geminatus* were identified in the GCF of RA patients. In addition, the taxon *P. gingivalis* displayed significant correlations with isotype IgA RFs, and was also further associated with more severe clinical presentations of RA. Taken together, our observations on detached GCF bacteria may provide new insights into associations between periodontitis and RA, and suggest that monitoring the bacterial composition of GCF might inform on RA activity and progression.

## AUTHOR CONTRIBUTIONS

Daniel Manoil contributed to data analysis, interpretation and drafted the manuscript. Nagihan Bostanci contributed to conception, design, data analysis, data interpretation, and critically revised the manuscript. Gonca Mumcu contributed to conception, design, data acquisition, interpretation and critically revised the manuscript. Nevsun Inanc contributed to conception, design, interpretation and critically revised the manuscript. Meryem Can contributed to design, data acquisition and critically revised the manuscript. Haner Direskeneli contributed to design, data interpretation and critically revised the manuscript. Georgios N. Belibasakis contributed to conception, design, data interpretation, and critically revised the manuscript. All authors gave final approval and agreed to be accountable for all aspects of the work.

## Supporting information

Supporting information.

Supporting information.

## Footnotes

* OraFlow Inc., Amityville, NY.

† GenericsAssays – GMBH, Berlin, DE.

‡ Protector RNase inhibitor, Sigma‐Aldrich, Buchs, Switzerland.

§ Adcell, Cel‐Line, Thermo‐Scientific, Reinach, Switzerland.

** OlympusBX60, Olympus Optical AG, Wallisellen, Switzerland.

†† Olympus E510, Olympus Optical AG, Wallisellen, Switzerland.
