# Supplementary material for: Novel and known periodontal pathogens residing in gingival crevicular fluid are associated with rheumatoid arthritis
Source: J Periodontol. 2020 Aug 27;92(3):359–70. doi: 10.1002/JPER.20-0295 (PMC8048861; doi:10.1002/JPER.20-0295)
Supplement: Supplementary file 1 — Supporting information. [file JPER-92-359-s001.docx]

**Supplementary table 1:** Oligonucleotide probes and antibodies (Ab) employed in this study.

| **Target taxon** | **Probe** | **Probe sequence (5’ 🡪 3’)** | **F %** | **Ref.** |
| --- | --- | --- | --- | --- |
| **Eubacteria** | EUB338 | GCT GCC TCC CGT AGG AGT | 40 | [^1^](#_ENREF_1)^,^ [^2^](#_ENREF_2) |
| ***P. intermedia*** | L-Pint649-2 | CGT TGC GTG CAC TCA AGT C | 40 | [^3^](#_ENREF_3) |
| ***Synergistetes* cl. A** | Syn-A-1409 | ACA CCC GGC TCG GGG GT | 40 | [^4^](#_ENREF_4)^,^ [^5^](#_ENREF_5) |
| ***Synergistetes* cl. B** | Syn-B-1149 | TCG ATG GCA GTC TCG CCG | 40 | [^6^](#_ENREF_6) |
| ***Leptotrichia*** | Lbuc668 | TAC TCG TGC AGT TCC GTC C | 40 | [^7^](#_ENREF_7) |
| ***A. geminatus*** | MegAg1147-Cy3 | TGC GGC WGT CTC TCC TGA | 40 | [^8^](#_ENREF_8) |
| ***Megasphaera*** | Meg1147-FAM | TGC GGC AGT CTC TCC TGA | 40 | [^9^](#_ENREF_9) |
| **TM7** | TM7-571 | CCR CCT ACG CAA CTC TTT AC | 40 | [^9^](#_ENREF_9) |
| **Target taxon** | **Ab name** | **Ab isotype** | **Ref** | |
| ***P. gingivalis*** | 61BG1.3 | Mouse IgG1 | [^10^](#_ENREF_10) | |
| ***T. forsythia*** | 103BF1.1 | Mouse IgG2b | [^11^](#_ENREF_11) | |
| **F**; formamide, **Ab**; antibody. | | | | |

**Oligonucleotide probes and antibodies references:**

1. Amann RI, Binder BJ, Olson RJ, Chisholm SW, Devereux R, Stahl DA. Combination of 16S rRNA-targeted oligonucleotide probes with flow cytometry for analyzing mixed microbial populations. *Appl Environ Microbiol* 1990;56:1919-1925.

2. Gmur R, Thurnheer T. Direct quantitative differentiation between *Prevotella intermedia* and *Prevotella nigrescens* in clinical specimens. *Microbiology* 2002;148:1379-1387.

3. Guggenheim B, Gmur R, Galicia JC, et al. In vitro modeling of host-parasite interactions: the 'subgingival' biofilm challenge of primary human epithelial cells. *BMC Microbiol* 2009;9:280.

4. Baumgartner JC, Falkler WA, Jr. Bacteria in the apical 5 mm of infected root canals. *J Endod* 1991;17:380-383.

5. Zijnge V, van Leeuwen MB, Degener JE, et al. Oral biofilm architecture on natural teeth. *PLoS One* 2010;5:e9321.

6. Baumgartner A, Thurnheer T, Luthi-Schaller H, Gmur R, Belibasakis GN. The phylum Synergistetes in gingivitis and necrotizing ulcerative gingivitis. *J Med Microbiol* 2012;61:1600-1609.

7. Gmur R, Wyss C, Xue Y, Thurnheer T, Guggenheim B. Gingival crevice microbiota from Chinese patients with gingivitis or necrotizing ulcerative gingivitis. *Eur J Oral Sci* 2004;112:33-41.

8. Bao K, Bostanci N, Thurnheer T, Belibasakis GN. Proteomic shifts in multi-species oral biofilms caused by *Anaeroglobus geminatus*. *Sci Rep* 2017;7:4409.

9. Lüdin ND. Phylogenetic characterization of subgingival plaque associated with aggressive or chronic periodontitis. Zürich, Switzerland: University of Zürich; 2011. 50 p.

10. Werner-Felmayer G, Guggenheim B, Gmur R. Production and characterization of monoclonal antibodies against *Bacteroides forsythus* and Wolinella recta. *J Dent Res* 1988;67:548-553.

11. Gmur R, Werner-Felmayer G, Guggenheim B. Production and characterization of monoclonal antibodies specific for *Bacteroides gingivalis*. *Oral Microbiol Immunol* 1988;3:181-186.
