# Supplementary material for: Novel and known periodontal pathogens residing in gingival crevicular fluid are associated with rheumatoid arthritis
Source: J Periodontol. 2020 Aug 27;92(3):359–70. doi: 10.1002/JPER.20-0295 (PMC8048861; doi:10.1002/JPER.20-0295)
Supplement: Supplementary file 2 — Supporting information. [file JPER-92-359-s002.docx]

**Supplementary table 2:** Comparison of the bacterial distribution between BD and HC groups (Mann-Whitney, p = 0.05).

| **Bacterial taxa** | **Groups** | **Median** | **SEM** | **Mean rank** | **p** |
| --- | --- | --- | --- | --- | --- |
| **Eubacteria** | **BD** | 2’145’000 | 4’241’598.4 | 59.03 | **0.003** |
|  | **HC** | 1’100’000 | 452’066.6 | 41.96 |  |
| ***P. gingivalis*** | **BD** | 1’160 | 58’871.7 | 54.71 | 0.092 |
|  | **HC** | 263 | 18’555 | 44.99 |  |
| ***P. intermedia*** | **BD** | 199 | 1870 | 53.13 | 0.153 |
|  | **HC** | 199 | 224.2 | 46.11 |  |
| ***T. forsythia*** | **BD** | 6’205 | 48’143.9 | 56.56 | **0.027** |
|  | **HC** | 405 | 16’401.8 | 43.69 |  |
| ***Synergistetes* cl. A** | **BD** | 263 | 10’425 | 52.4 | 0.318 |
|  | **HC** | 300 | 6’313.7 | 46.61 |  |
| ***Synergistetes* cl. B** | **BD** | 29 | 194.4 | 62.01 | **0.000** |
|  | **HC** | 29 | 60.2 | 39.87 |  |
| ***Leptotrichia*** | **BD** | 219 | 4’314.8 | 54.01 | 0.14 |
|  | **HC** | 113 | 1’369.2 | 45.48 |  |
| ***A. geminatus*** | **BD** | 87 | 52.5 | 65.16 | **0.000** |
|  | **HC** | 29 | 8.4 | 37.66 |  |
| ***Megasphaera*** | **BD** | 150 | 20 | 62.08 | **0.000** |
|  | **HC** | 29 | 49.7 | 39.82 |  |
| **TM7** | **BD** | 29 | 2.1 | 57.95 | **0.000** |
|  | **HC** | 29 | 2.8 | 41.75 |  |
| Significant p values are bolded. **RA**; rheumatoid arthritis, **BD** Behçet disease, **HC**; healthy control, **SEM**; standard error of the mean. | | | | | |
